# Molecular Detection of *Candidatus* Orientia chuto in Wildlife, Saudi Arabia

**DOI:** 10.3201/eid2902.221131

**Published:** 2023-02

**Authors:** Hadil A. Alkathiry, Samia Q. Alghamdi, Holly E.J. Morgan, Madeleine E. Noll, Jing J. Khoo, Abdulaziz N. Alagaili, Benjamin L. Makepeace

**Affiliations:** Al Imam Mohammad Ibn Saudi Islamic University, Riyadh, Saudi Arabia (H.A. Alkathiry);; University of Liverpool, Liverpool, England, UK (H.A. Alkathiry, H.E.J. Morgan, M.E. Noll, J.J. Khoo, B.L. Makepeace);; Al-Bahah University, Al-Bahah, Saudi Arabia (S.Q. Alghamdi); King Saud University, Riyadh (A.N. Alagaili)

**Keywords:** scrub typhus, bacteria, Candidatus Orientia chuto, zoonoses, vector-borne infections, parasites, Hijaz, Asir, Al-Bahah, chigger, Acomys, Dipodillus, Saudi Arabia

## Abstract

Scrub typhus is a zoonosis caused by 3 species of *Orientia* bacteria, including *Candidatus* Orientia chuto. This species is known only from a human case in Dubai and infections in wildlife in Kenya. We report molecular detection of *Candidatus* O. chuto in 2 wild rodent species from Saudi Arabia.

Scrub typhus is a zoonotic bacterial disease caused by 3 intracellular species of bacteria in the genus *Orientia* (Rickettsiales: Rickettsiaceae). The disease is widespread in the Asia-Pacific Region and is associated with fever, as well as pneumonitis, encephalitis, and myocarditis if not promptly treated. The median case fatality rate is ≈6% (*1*), and one third of infections during pregnancy result in adverse outcomes (*2*). 

*Orientia* spp. are transmitted to humans through the bite of infected trombiculid mite larva (chiggers), which feed primarily on small mammals or birds and only incidentally attack humans. *Orientia* spp. are maintained by vertical transmission in trombiculid mites, but wild vertebrate hosts can become infected. However, whether host species are epidemiologically meaningful *Orientia* reservoirs is controversial because horizontal transmission of *Orientia* spp. to chiggers during feeding rarely translates into successful transstadial transfer and transovarial transmission into the next generation (*3*). At minimum, wild hosts contribute to scrub typhus risk by amplifying trombiculid populations; individual hosts can potentially be infested with thousands of chiggers simultaneously (*4*).

Until the early 21st Century, only 1 species of *Orientia* was known: *Orientia tsutsugamushi*, which is restricted to the tsutsugamushi triangle across the Asia-Pacific region. The precise western limit of *O. tsutsugamushi* endemicity is unclear, but reports beyond the Hindu Kush region, where Afghanistan, Tajikistan, and Pakistan converge, are very rare. However, in 2006, a woman contracted scrub typhus while visiting Dubai (*5*). The pathogen was isolated in culture, and molecular characterization established that it was sufficiently distinct from *O. tsutsugamushi* to be classified as a new species, *Candidatus* Orientia chuto. Since that report, *Candidatus* O. chuto–like sequences rarely have been detected, but 1 report describes detection from chiggers infesting a Natal multimammate mouse (*Mastomys natalensis*) in Baringo County, Kenya (*6*). A third species of scrub typhus pathogen, *Candidatus* O. chiloensis, was recently described from patients in Chile (*7*). We investigated whether *Orientia* spp. are circulating in small mammals and chiggers in Saudi Arabia.

## The Study

**Figure 1:**
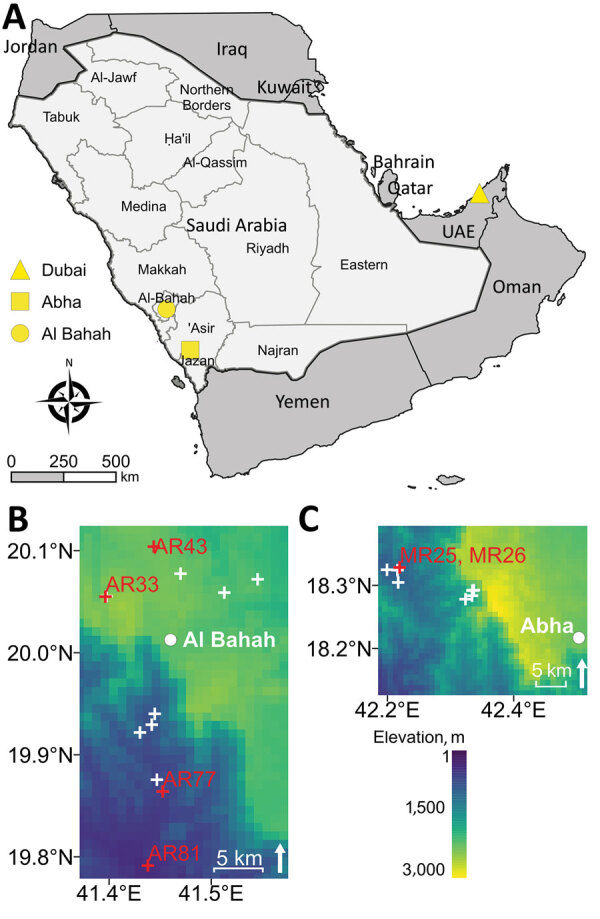
Sampling sites and elevation from which rodents were collected for molecular detection of *Candidatus* Orientia chuto in wildlife, Saudi Arabia. A) Study region on the Arabian Peninsula, including Dubai (yellow triangle), where a clinical case of scrub typhus caused by *Candidatus* O. chuto was reported in a previous study (*5*). Light gray area indicates Saudi Arabia; dark gray area indicates bordering countries on the Arabian Peninsula. Rodents were trapped in the Hijaz Mountains and surrounding towns of Al-Bahah Province (yellow circle indicates Al-Bahah, the capital city) and in the Asir Mountains of Asir Province (yellow square indicates Abha, the capital city). B, C) Heat maps detailing elevation above sea level of trapping locations in Al-Bahah Province (B) and Asir Province (C). Red crosses and sample labels indicate where *Orientia*-positive rodents were found; white crosses indicate areas in which rodents showed no evidence of infection. UAE, United Arab Emirates.

We trapped rodents in southwestern Saudi Arabia, as previously described (*8*) (Figure 1). We humanely euthanized rodents by inhaled anesthetic overdose and preserved any attached chiggers in 70% ethanol. The fieldwork was approved by the Saudi Wildlife Authority (approval no. 288/33/A) and the University of Liverpool’s Animal Welfare and Ethics Review Board. 

We dissected rodents and fixed the internal organs (lungs, spleen, liver, and kidney) in 70% ethanol. To identify chiggers, we cleared a 10% subsample from each rodent in Berlese fluid and mounted these for microscopic examination (*8*). We pooled the remaining chiggers by species (10–30 specimens per pool) from each rodent. We crushed chigger pools with a pellet-pestle, then extracted DNA by using the DNeasy Blood & Tissue Kit (QIAGEN, https://www.qiagen.com) according to the manufacturer’s instructions and eluted DNA in 30 μL ultrapure water. 

We purified genomic DNA from individual mammal host organs (10 mg spleen or 25 mg for other organs) by using the DNeasy Blood & Tissue Kit (QIAGEN) and eluting DNA in 50 μL ultrapure water. We identified rodents by amplifying a cytochrome B gene fragment and performing BLAST analysis (https://blast.ncbi.nlm.nih.gov). 

To detect *Orientia* DNA, we screened rodent and chigger extracts by using a quantitative PCR targeting the multicopy *traD* gene (*9*). We subjected positive samples to nested PCRs designed to amplify the 47 kDa high-temperature requirement A (*htrA*) gene*,* also known as TSA47, from *O. tsutsugamushi* or *Candidatus* O. chuto (*6*). We sent amplicons from the second round of the nested PCR to Eurofins Genomics (https://www.eurofins.com) for Sanger sequencing in both directions, then trimmed results to 698 bp, and aligned with reference sequences from GenBank. We used MrBayes (*10*) to construct a phylogenetic tree by using MUSCLE alignment in the Phylogeny.fr web service (*11*). We estimated the best-fit model of nucleotide substitution by using Akaike information criterion in jModelTest 2.1.7 (*12*) and selected the general time-reversible plus gamma distribution plus invariable site model as the best fit. We calculated pairwise distances between sequences by using MEGA 11 (https://www.megasoftware.net).

We trapped 27 rodents in Asir Province, all of which we identified as Eastern spiny mice (*Acomys dimidiatus*). We trapped 55 rodents of 3 different species in Al-Bahah Province (Table 1). Using *htrA* primers for *Candidatus* O. chuto, we identified 7 sequence-confirmed positive organs from 6 individual animals, 2 (7.4%) from Asir Province and 4 (7.3%) from Al-Bahah Province (Table 2). We found infected *A. dimidiatus* in both provinces, and a single infected Wagner’s gerbil (*Dipodillus dasyurus*) in Al-Bahah. Positive rodents were widely distributed both in terms of habitat type and elevation (388–2,477 meters above sea level) at the trap location (Table 2; Figure 1). One *A. dimidiatus* from Asir had 2 organs that tested positive for *Candidatus* O. chuto, but all other rodents had a single PCR-positive organ (Table 2). Only 2 rodents were infested with chiggers, and we identified a total of 5 chigger species. None of the chigger samples were PCR-positive for *Orientia* spp. DNA.

**Table 1:** Wild rodents screened during molecular detection of *Candidatus* Orientia chuto in wildlife, Saudi Arabia*

Common name	Latin name	Province	
		Asir	Al-Bahah
Eastern spiny mouse	*Acomys dimidiatus*	27	48
Wagner’s gerbil	*Dipodillus dasyurus*	0	4
House mouse	*Mus musculus*	0	3
Total		27	55

*Rodents were identified by amplification of a fragment of the cytochrome B gene and sequences were uploaded to the Barcode of Life Data System version 4 (https://boldsystems.org), under project code HAK.

**Table 2:** Positive rodent specimens, chigger infestations, and collection sites used for molecular detection of *Candidatus* Orientia chuto in wildlife, Saudi Arabia

Province, sample ID	Host species	Tissue, *htrA* accession no.	No. chiggers per species*	Nearest settlement or feature	GPS coordinates	Elevation†	Collection date
Asir							Oct 2020
MR25	*Acomys dimidiatus*	Liver, ON844109	12 *E. caucasicum*; 16 *E. kazeruni*; 1 *S. saudi*; 1 *S. zarudnyi*	Wosanib village	N18.328347, E42.219233	917	
MR26	*A. dimidiatus*	Liver, ON84411; kidney, ON844111	None	Wosanib village	N18.328347, E42.219233	917	
Al-Bahah							Aug 2021
AR33	*Dipodillus dasyurus*	Spleen, ON844112	1 *M. hoogstraali*	Khairah Forest Park	N20.054973, E41.396330	2,477	
AR43	*A. dimidiatus*	Spleen, ON844113	None	Bani Sar town	N20.103946, E41.443762	2,218	
AR77	*A. dimidiatus*	Spleen, ON844114	None	King Fahd Road, Al Makhwah	N19.864027, E41.452468	545	
AR81	*A. dimidiatus*	Spleen, ON844115	None	King Fahd Road, Al Makhwah	N19.791421, E41.437912	388	


*Full genus names: *Ericotrombidium*, *Schoutedenichia*, *Microtrombicula*. †Meters above sea level.

**Figure 2:**
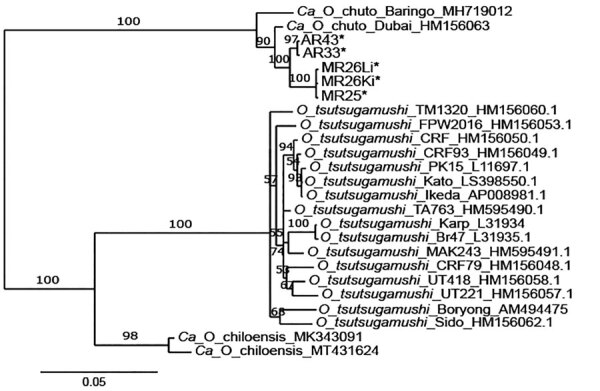
Bayesian inference phylogenetic tree of *Orientia htrA* gene sequences detected in wildlife, Saudi Arabia. Tree represents 698 nt positions of *hrtA*. Asterisks (*) indicate samples from this study; identification numbers are listed in Table 2. Only sequences confirmed by 2 independent PCR amplifications of the same tissue are included. Numbers at nodes represent Bayesian posterior probabilities (%). Accession numbers are given for reference sequences from GenBank. Ki, kidney; Li, liver.

Phylogenetic analysis of publicly available *htrA* gene sequences revealed 3 well-supported clades representing *O. tsutsugamushi*, *Candidatus* O. chiloensis, and *Candidatus* O. chuto (Figure 2). The *Candidatus* O. chuto lineage was split into 2 main clades comprising isolates from the Arabian Peninsula, including isolates from this study and the clinical isolate from Dubai in 1 clade and the isolate from Kenya in the other clade. The *Candidatus* O. chuto sequences from this study were distinct from the Dubai isolate (pairwise distance of 0.7%–1.3%) and comprised 2 genotypes exhibiting a pairwise distance of 0.9% (Appendix Table), which was linked to geographic origin from Al-Bahah or Asir Province (Figure 2).

## Conclusions

Only 2 published records of *Candidatus* O. chuto have been available worldwide, 1 from Dubai (*5*) and 1 from Baringo, Kenya (*6*), and only the Dubai isolate has been cultured in vitro and fully sequenced. We identified 2 *Orientia* genotypes from Saudi Arabia, neither of which was identical to previously described isolates. Separation between *Orientia* spp. at the *htrA* locus is ≈11%–14%, whereas within *O. tsutsugamushi*, *htrA* attains a maximum separation of 1.5%. The maximum separation between the sequences from the Arabian Peninsula and Kenya was 2.5% (Appendix Table), indicating that whole-genome sequencing studies are needed to resolve relationships between *Candidatus* O. chuto–like organisms. 

The potential range of *Candidatus* O. chuto is vast, and the bacterium is suspected of causing scrub typhus on the continent of Africa, which is indicated by serologic and clinical evidence, albeit without confirmation by sequencing or culture to date. The *Orientia*-infected rodents detected in this study were found in locations >2,000 km northeast of Baringo County, Kenya, and >1,500 km southwest of Dubai. Thus, the endemic zone of *Candidatus* O. chuto clearly includes northeastern Africa and the Arabian Peninsula, at least, but also could potentially extend eastwards to the western extremity of the tsutsugamushi triangle. Of note, *Orientia* spp. detected by 16S amplicon sequencing in house mice (*Mus musculus domesticus*) in West Africa resembled *Candidatus* O. chuto, although sequences obtained from rodents in France formed a clade that was distinct from known *Orientia* spp. (*13*).

Scrub typhus has not been reported from Saudi Arabia, but our findings highlight the need for vigilance. Moreover, further investigations of the diverse chigger fauna of Saudi Arabia are required to identify the local vectors of *Orientia* spp. Because other agents of febrile illness are endemic in the southwest of the country, including *Plasmodium vivax* (*14*) and dengue virus (*15*), and the region is popular with tourists because of its relatively cool climate, we recommend scrub typhus be included in the differential diagnosis for fever in Saudi Arabia. 

Appendix Pairwise distances between *Orientia* spp. *htrA* sequences molecularly detected in a study of *Candidatus* Orientia chuto in wildlife, Saudi Arabia.

## References

[1] Bonell A, Lubell Y, Newton PN, Crump JA, Paris DH. Estimating the burden of scrub typhus: A systematic review. PLoS Negl Trop Dis. 2017;11:e0005838. 10.1371/journal.pntd.0005838 28945755 PMC5634655

[2] McGready R, Prakash JAJ, Benjamin SJ, Watthanaworawit W, Anantatat T, Tanganuchitcharnchai A, et al. Pregnancy outcome in relation to treatment of murine typhus and scrub typhus infection: a fever cohort and a case series analysis. PLoS Negl Trop Dis. 2014;8:e3327. 10.1371/journal.pntd.0003327 25412503 PMC4238995

[3] Traub R, Wisseman CL Jr, Jones MR, O’Keefe JJ. The acquisition of *Rickettsia tsutsugamushi* by chiggers (trombiculid mites) during the feeding process. Ann N Y Acad Sci. 1975;266(1 Pathobiology):91–114. 10.1111/j.1749-6632.1975.tb35091.x 829479

[4] Elliott I, Pearson I, Dahal P, Thomas NV, Roberts T, Newton PN. Scrub typhus ecology: a systematic review of *Orientia* in vectors and hosts. Parasit Vectors. 2019;12:513. 10.1186/s13071-019-3751-x 31685019 PMC6829833

[5] Izzard L, Fuller A, Blacksell SD, Paris DH, Richards AL, Aukkanit N, et al. Isolation of a novel *Orientia* species (*O. chuto* sp. nov.) from a patient infected in Dubai. J Clin Microbiol. 2010;48:4404–9. 10.1128/JCM.01526-10 20926708 PMC3008486

[6] Masakhwe C, Linsuwanon P, Kimita G, Mutai B, Leepitakrat S, Yalwala S, et al. Identification and characterization of *Orientia chuto* in trombiculid chigger mites collected from wild rodents in Kenya. J Clin Microbiol. 2018;56:e01124–18. 10.1128/JCM.01124-18 30282787 PMC6258837

[7] Abarca K, Martínez-Valdebenito C, Angulo J, Jiang J, Farris CM, Richards AL, et al. Molecular description of a novel *Orientia* species causing scrub typhus in Chile. Emerg Infect Dis. 2020;26:2148–56. 10.3201/eid2609.200918 32818400 PMC7454112

[8] Stekolnikov AA, Al-Ghamdi SQ, Alagaili AN, Makepeace BL. First data on chigger mites (Acariformes: Trombiculidae) of Saudi Arabia, with a description of four new species. Syst Appl Acarol. 2019;24:1937–63. 10.11158/saa.24.10.12

[9] Chao C-C, Belinskaya T, Zhang Z, Jiang L, Ching W-M. Assessment of a sensitive qPCR assay targeting a multiple-copy gene to detect *Orientia tsutsugamushi* DNA. Trop Med Infect Dis. 2019;4:113. 10.3390/tropicalmed4030113 31370347 PMC6789807

[10] Ronquist F, Teslenko M, van der Mark P, Ayres DL, Darling A, Höhna S, et al. MrBayes 3.2: efficient Bayesian phylogenetic inference and model choice across a large model space. Syst Biol. 2012;61:539–42. 10.1093/sysbio/sys029 22357727 PMC3329765

[11] Dereeper A, Guignon V, Blanc G, Audic S, Buffet S, Chevenet F, et al. Phylogeny.fr: robust phylogenetic analysis for the non-specialist. Nucleic Acids Res. 2008;36(Suppl 2):W465-9. 10.1093/nar/gkn180 18424797 PMC2447785

[12] Posada D. jModelTest: phylogenetic model averaging. Mol Biol Evol. 2008;25:1253–6. 10.1093/molbev/msn083 18397919

[13] Cosson JF, Galan M, Bard E, Razzauti M, Bernard M, Morand S, et al. Detection of *Orientia* sp. DNA in rodents from Asia, West Africa and Europe. Parasit Vectors. 2015;8:172. 10.1186/s13071-015-0784-7 25884521 PMC4374543

[14] Al-Mekhlafi HM, Madkhali AM, Ghailan KY, Abdulhaq AA, Ghzwani AH, Zain KA, et al. Residual malaria in Jazan region, southwestern Saudi Arabia: the situation, challenges and climatic drivers of autochthonous malaria. Malar J. 2021;20:315. 10.1186/s12936-021-03846-4 34256757 PMC8276496

[15] Alhaeli A, Bahkali S, Ali A, Househ MS, El-Metwally AA. The epidemiology of Dengue fever in Saudi Arabia: A systematic review. J Infect Public Health. 2016;9:117–24. 10.1016/j.jiph.2015.05.006 26106040

